# Correlation Between Volumetric Soft Tissue Asymmetry and Postero-Anterior Cephalometric Measurements in Patients with Skeletal Facial Asymmetry: A Cross-Sectional Pilot Study

**DOI:** 10.3390/jcm14196721

**Published:** 2025-09-23

**Authors:** Saki Tanaka, Yudai Shimpo, Hiromi Sato, Toshiko Sekiya, Shotaro Ueda, Chihiro Kariya, Takashi Oikawa, Hiroshi Tomonari

**Affiliations:** Department of Orthodontics, Tsurumi University School of Dental Medicine, Yokohama 230-8501, Japan; tnk.saki.0321@gmail.com (S.T.); sato-h1112@outlook.jp (H.S.); sekiya-t@tsurumi-u.ac.jp (T.S.); 0508shou@gmail.com (S.U.); chihirortho.k@gmail.com (C.K.); oikawa-t@tsurumi-u.ac.jp (T.O.); tomonari-h@tsurumi-u.ac.jp (H.T.)

**Keywords:** facial asymmetry, postero-anterior cephalogram, three-dimensional imaging, orthognathic surgery, stereophotogrammetry, facial scanner, cephalometric analysis

## Abstract

**Background/Objectives:** While skeletal facial asymmetry is commonly assessed using posteroanterior (PA) cephalometric radiographs, the association between skeletal measurements and volumetric soft tissue asymmetry remains unclear. This study aimed to identify which skeletal parameters are most strongly correlated with soft tissue asymmetry measured using three-dimensional (3D) imaging. **Methods:** Thirty-three Japanese patients (8 males and 25 females; mean age: 26.85 ± 12.13 years) undergoing orthodontic–orthognathic treatment were included. Three-dimensional facial surface data were acquired using the VECTRA^®^ H1 imaging system. Soft tissue asymmetry was quantified by calculating the volumetric difference between the original and mirrored 3D facial images, divided into three regions: whole face, midface, and lower face. PA cephalometric radiographs were traced, and 28 skeletal variables were measured. Pearson correlation coefficients were calculated between skeletal variables and asymmetry volumes and squared to obtain R^2^ values. **Results:** The strongest correlation with whole facial soft tissue asymmetry was found for menton deviation from the midline (R^2^ = 0.630). Similar trends were observed for the lower face. In contrast, only one skeletal variable showed a moderate correlation with midfacial asymmetry (maximum R^2^ = 0.186), and skeletal parameters related to maxillary occlusal cant did not show significant associations. **Conclusions:** Volumetric soft tissue asymmetry is strongly associated with mandibular skeletal deviation, particularly menton displacement, whereas midfacial skeletal morphology may have a limited impact. Further studies including more patients with pronounced midfacial soft tissue asymmetry are warranted.

## 1. Introduction

Facial symmetry is considered one of the key elements of facial attractiveness and is often associated with perceived health, youthfulness, and functional balance [1,2]. In clinical settings, facial asymmetry—defined as a lack of proportional harmony between the left and right sides of the face—poses both esthetic and functional challenges [3,4]. While slight asymmetries are common in the general population and often go unnoticed, moderate to severe skeletal asymmetries may contribute to malocclusion, temporomandibular joint disorders (TMD), and psychosocial distress [5,6,7]. Therefore, accurate assessment and characterization of facial asymmetry are important for diagnosis, treatment planning, and outcome evaluation in orthodontics and orthognathic surgery [2,8].

Skeletal facial asymmetry is commonly assessed using postero-anterior (PA) cephalometric radiographs, which allow for the quantification of transverse skeletal discrepancies such as chin deviation, mandibular ramus height differences, and maxillary cant [9,10]. Numerous studies have analyzed skeletal landmarks on PA radiographs to classify the type and severity of asymmetry and to assist in planning corrective interventions [11,12]. These radiographic analyses have traditionally focused on hard tissue structures, assuming that soft tissue contours reflect the underlying skeletal framework [13,14]. However, recent advances in three-dimensional (3D) imaging techniques have challenged this assumption by enabling direct, volumetric assessment of soft tissue morphology, overcoming some inherent limitations of conventional two-dimensional cephalometric radiographs, such as image distortion and the superimposition of anatomical structures that can obscure landmarks [15,16,17,18].

In contrast to conventional two-dimensional imaging, 3D stereophotogrammetry provides a non-invasive, radiation-free modality for capturing high-resolution facial surface data [19,20,21]. It enables objective quantification of soft tissue asymmetry through techniques such as mirroring and superimposition, which allow for the measurement of volumetric differences between the original face and its mirror image [22,23,24]. These 3D methods offer a more accurate representation of facial contour and esthetic perception, which are ultimately shaped by soft tissues rather than underlying bone alone [25]. Despite the increasing use of 3D imaging in clinical practice, there are relatively few reports, and the relationship between skeletal asymmetry and overlying soft tissue imbalance remains far from fully understood [26,27].

Specifically, the extent to which skeletal discrepancies detected on PA cephalograms influence volumetric soft tissue asymmetry is not well established. While some studies have reported that mandibular deviation, particularly menton displacement from the facial midline, is closely associated with lower facial asymmetry [8], findings regarding midfacial asymmetry are less consistent. Some authors have suggested that midfacial skeletal structures, such as maxillary width or occlusal cant, may contribute to midface soft tissue asymmetry [28]. Others argue that the midface exhibits greater soft tissue compensation or masking due to its anatomical complexity and thicker soft tissue coverage, thus showing weaker associations with skeletal landmarks [29].

Furthermore, few studies have attempted to quantify the relative contributions of skeletal features to regional soft tissue asymmetry using volumetric analysis [30]. Most existing research focuses on linear or angular asymmetry indices and rarely distinguishes between different facial regions [31]. As a result, it remains unclear which cephalometric variables are most predictive of asymmetry in the midface or lower face and whether the same skeletal determinants are applicable across facial regions.

To address this gap, the present study aimed to investigate the correlation between skeletal asymmetry parameters measured on PA cephalograms and volumetric soft tissue asymmetry obtained via 3D stereophotogrammetry. By evaluating the whole face, midface, and lower face separately, we sought to identify which skeletal landmarks are most closely associated with soft tissue imbalance. Particular attention was given to distinguishing the skeletal influences on midfacial asymmetry from those affecting the lower face, as this distinction has significant implications for both diagnosis and treatment planning.

We hypothesized that lower facial skeletal deviations, such as menton displacement and mandibular body asymmetry, would show strong correlations with volumetric soft tissue asymmetry in the lower face. In contrast, we expected that midfacial skeletal variables—such as maxillary cant or nasal septum deviation—would exhibit weaker or inconsistent associations with midfacial soft tissue asymmetry. Understanding these region-specific relationships may help refine clinical assessment protocols and improve the predictability of soft tissue outcomes following orthognathic surgery.

## 2. Materials and Methods

### 2.1. Subjects, Eligibility Criteria and Ethics

This cross-sectional study included 33 Japanese patients (8 males and 25 females; mean age ± SD: 26.85 ± 12.13 years) diagnosed with skeletal facial asymmetry and scheduled to undergo orthognathic surgery at the Department of Orthodontics, Tsurumi University Dental Hospital. All participants were examined by the same experienced orthodontist and diagnosed as having facial asymmetry based on clinical examination and posteroanterior (PA) cephalometric analysis, regardless of anteroposterior skeletal classification (Class I, II, or III). Inclusion and exclusion criteria were determined with reference to previous studies [28,30,32].

Inclusion criteria were as follows:

(1) Japanese adults and adolescents with permanent dentition.
(2) Diagnosis of facial asymmetry by both clinical assessment and radiographic evaluation.
(3) Planned orthognathic surgery as part of treatment at our institution.
(4) No history of orthodontic or orthognathic treatment prior to enrollment.

Exclusion criteria included:

(1) Presence of craniofacial syndromes (e.g., hemifacial microsomia), congenital anomalies such as cleft lip and/or palate.
(2) History of facial trauma or maxillofacial surgery that could alter craniofacial morphology.
(3) Systemic diseases or conditions that could affect craniofacial growth and development.

This retrospective study was initially approved by the Institutional Review Board of Tsurumi University School of Dental Medicine on 3 August 2020 (Approval No. 1810). Due to minor modifications, including changes in the researchers’ positions and academic roles within the university as well as administrative updates, an expedited review was subsequently conducted, and the updated protocol received approval on 25 February 2025 (Approval No. 124022).

### 2.2. Three-Dimensional Facial Image Acquisition and Asymmetry Assessment

Three-dimensional facial photographs were obtained using a handheld stereophotogrammetry system (VECTRA^®^ H1, Canfield Scientific, Parsippany, NJ, USA), following the protocols described in previous studies [33,34,35]. All image acquisitions were performed by a single calibrated examiner. Participants were instructed to maintain a natural head position, with the Frankfurt horizontal plane parallel to the floor, eyes gazing forward, lips gently closed, and facial muscles relaxed. Hair, clothing, and accessories were positioned so as not to obscure the face, ears, or neck.

Following the manufacturer’s guidelines, three sequential stereophotographs were taken for each participant: (1) a frontal view with the green targeting lights converged on the philtrum, (2) a 45° left oblique view with the lights converged on the center of the left cheek, and (3) a 45° right oblique view with the lights converged on the center of the right cheek. The built-in targeting system ensured consistent camera–subject distance and orientation. These three images were automatically stitched into a single 3D facial surface using Mirror^®^ software, version 7.4 (Canfield Scientific, Parsippany, NJ, USA).

**Figure 1:**
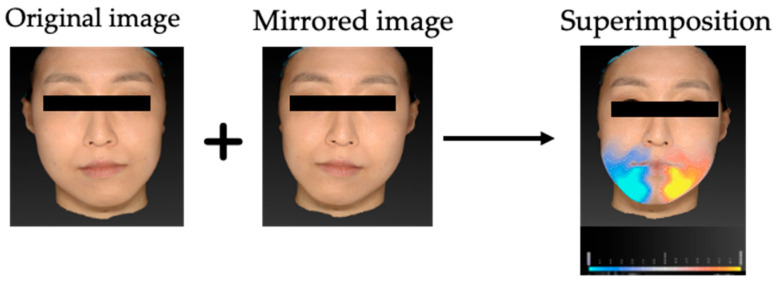
Process of volumetric asymmetry assessment using 3D stereophotogrammetry. The original 3D facial surface was mirrored across the constructed midsagittal plane, and the mirrored surface was superimposed on the original using a best-fit algorithm. The absolute volumetric difference between the original and mirrored surfaces was calculated for each region of interest.

For soft tissue asymmetry assessment, each 3D facial model was mirrored across the constructed midsagittal plane and superimposed on the original surface using a best-fit algorithm. The midsagittal plane was defined in accordance with Ueda et al. [35], by constructing a plane perpendicular to the coronal plane through the sellion (Se) and subnasale (Sn). This definition ensured anatomical consistency across participants. Previous validation studies demonstrated that the VECTRA^®^ H1 system provides high measurement accuracy, repeatability, and reproducibility (coefficients of variation typically < 3%), supporting the reliability of midline-based assessments [33,34]. Intra- and inter-operator errors for linear measurements using this system are reported to be within 1 mm, which is considered clinically acceptable [33,34]. The absolute volumetric difference between the original and mirrored surfaces was calculated for each region of interest (Figure 1). The facial surface was divided into three regions based on planes parallel to the Frankfort horizontal (FH) plane, following the method described in previous studies [34,35,36]:

Midface: the region between two planes parallel to the FH plane passing through the exocanthion and the cheilion.

Lower face: the region between two planes parallel to the FH plane passing through the cheilion and the menton.

Whole face: the combined area of the midface and lower face regions.

In all regions, the nasal and supraorbital areas above the eyes were excluded from measurement. The primary outcome measures were the absolute volumetric differences (cm^3^) for each region (whole face, midface, and lower face).

### 2.3. Cephalometric Analysis

Postero-anterior (PA) cephalograms were obtained with ear rods using a CX-150ST 8000C device (ASAHIROENTGEN IND. Co., Ltd., Kyoto, Japan) under the following settings: 150 kV, 250 mA, 0.32 s exposure time, and a focus–film distance (FFD) of 1650 mm. All radiographs were taken by a single examiner with the patient’s head positioned so that the Frankfurt horizontal plane was parallel to the floor and the midsagittal plane was perpendicular to the X-ray beam. These images were acquired by a certified radiologic technologist at the Department of Diagnostic Imaging, Tsurumi University Dental Hospital.

**Figure 2:**
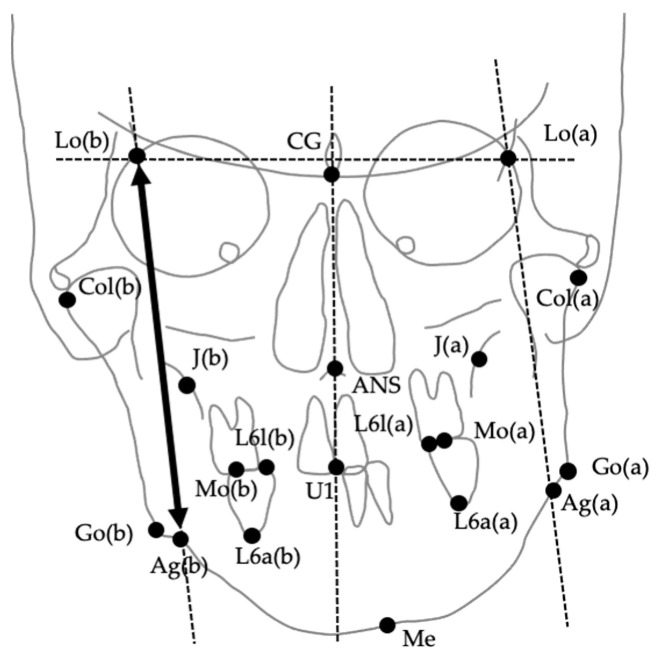
Anatomical landmarks used for posteroanterior (PA) cephalometric analysis. (a) Deviated side; (b) Contralateral side. Landmarks include CG (crista galli), ANS (anterior nasal spine), Lo (intersection of the orbital rim and oblique line), J (intersection of the maxillary tuberosity and zygomatic arch), Mo (upper first molar), U1 (maxillary incisor midline), Go (gonion), Ag (antegonial notch), and Me (menton).

**Figure 3:**
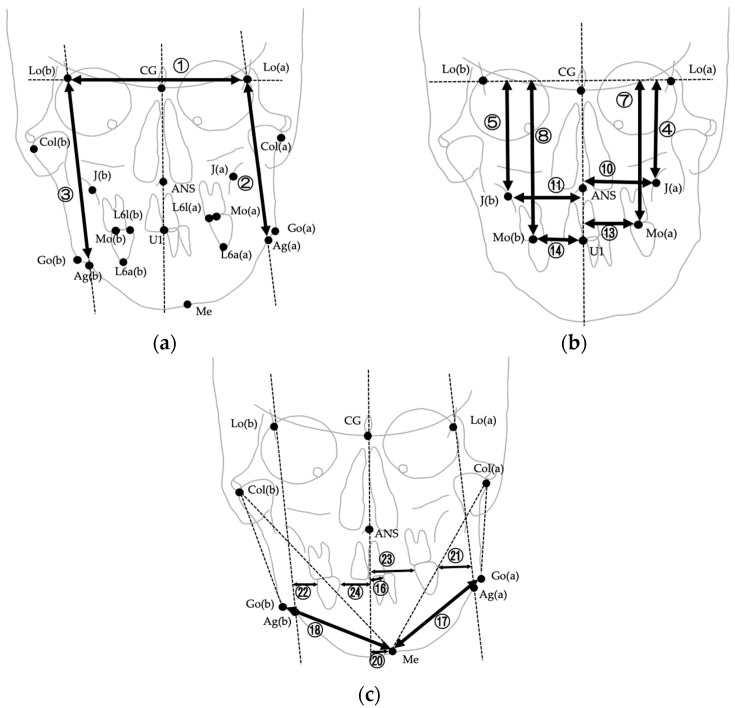
Linear measurements used in PA cephalometric analysis. Numbers correspond to the measurement items listed in Table 1. (**a**) Measurements ①–③ (Lo line, FP(a), FP(b)); (**b**) Measurements ④–⑭ (Lo line–J, Lo line–Mo, Midline–J, Midline–Mo, and related measurements except for differences); (**c**) Measurements ⑯–㉔ (Midline–U1, Go–Me, Midline–Me, and upper first molar to FP and midline). Please note that difference measurements (⑥, ⑨, ⑫, ⑮, ⑲) are defined in Table 1 but not illustrated in this figure.

**Figure 4:**
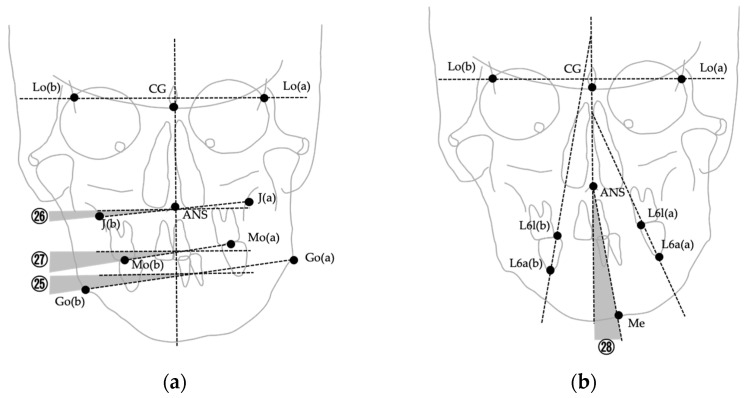
Angular measurements used in PA cephalometric analysis. Numbers correspond to the measurement items listed in Table 1. (**a**) Measurements ㉕–㉗ (∠Fmp, ∠J, ∠Ocl); (**b**) Measurement ㉘ (∠Mea, angle between ANS–Me and the midline).

The images were digitized and analyzed using WinCeph ver. 11.0 (Rise Co., Ltd., Sendai, Japan). Twenty-eight cephalometric measurements were performed based on anatomical landmarks and reference planes commonly used in previous studies [8,29,37,38,39]. The midsagittal reference line was defined by connecting the crista galli and the maxillary incisor midpoint (U1), following established PA cephalometric protocols for facial asymmetry assessment [38,39]. This approach has been widely adopted to ensure consistency across studies; however, it should be noted that in cases with inherent maxillary skeletal asymmetry, the use of U1 as a reference point may partially mask discrepancies between skeletal and soft tissue midfacial structures. The selected variables included linear and angular parameters describing transverse skeletal relationships, mandibular deviation, maxillary cant, and asymmetry indices. Landmarks, definitions, and measurement methods are summarized in Table 1, and representative examples are illustrated in Figure 2, Figure 3 and Figure 4. Figure 2 shows the landmarks used for PA cephalometric analysis (a: deviated side, b: contralateral side). Figure 3a–c present the linear measurement items on PA cephalograms, and Figure 4a,b depict the angular measurement items. The choice of these 28 parameters was guided by established cephalometric protocols for facial asymmetry assessment, ensuring consistency with earlier research while enabling detailed quantitative evaluation of skeletal morphology relevant to soft tissue asymmetry.

### 2.4. Reliability

To assess intra-examiner reliability, all measurements for both the 3D stereophotogrammetric images and PA cephalograms were performed by a single experienced examiner. For reliability testing, 10 patients were randomly selected from the study sample. For the PA cephalometric analysis, all 28 cephalometric variables were re-traced and re-measured on the selected radiographs two weeks after the initial measurements. Similarly, for the 3D facial images acquired using the VECTRA^®^ H1 system, landmark digitization and volumetric asymmetry calculations for all three facial regions (whole face, midface, lower face) were repeated after the same two-week interval.

No reference to the previous measurements was allowed during the second assessment to avoid bias. For each variable, the intraclass correlation coefficient (ICC, two-way mixed-effects model, absolute agreement) was calculated to quantify intra-examiner reliability. The ICC values were interpreted according to the guidelines of Landis and Koch (1977) [40]: <0.00 = poor, 0.00–0.20 = slight, 0.21–0.40 = fair, 0.41–0.60 = moderate, 0.61–0.80 = substantial, and 0.81–1.00 = almost perfect agreement [40].

In addition, the technical error of measurement (TEM) was computed using Dahlberg’s formula:

TEM = ∑d22nTEM = 2n∑d2

where dd is the difference between the two measurements and nn is the number of paired observations. The coefficient of reliability (R) was also calculated as:

R = 1 − TEM2σ2R = 1 − σ2TEM2

where σ2σ2 is the variance of the measurements. For TEM, smaller values indicated higher measurement precision, while R values closer to 1 denoted higher reliability.

The results demonstrated excellent intra-examiner reliability for both 3D stereophotogrammetric and PA cephalometric measurements, with ICC values in the “almost perfect” range for all variables. TEM values were small across all parameters, and R values were consistently high, indicating that measurement error was negligible. These findings are consistent with previous reports on the reproducibility of VECTRA^®^ H1 imaging [29,33,34,38] and PA cephalometric measurements in facial asymmetry assessment [30].

### 2.5. Sample Size Calculation

A priori sample size estimation was performed using G*Power software, version 3.1 (Heinrich Heine University Düsseldorf, Düsseldorf, Germany) (two-tailed Exact test, Correlation: Bivariate normal model). Parameters were set as follows: expected correlation under the alternative hypothesis ρH1 = 0.50 (Cohen’s convention effect [41]), significance level α = 0.05, desired power 1 – β = 0.80, and null hypothesis correlation ρH0 = 0. Under these settings, the required sample size was *n* = 29. Our final sample (*n* = 33) met and exceeded this requirement. The two-tailed specification reflects bidirectional testing; the effect size choice (r = 0.50) followed Cohen’s benchmarks for correlations [42].

### 2.6. Statistical Analysis

Descriptive statistics (mean, standard deviation, minimum, maximum) were calculated for all variables. The normality of the data distribution was assessed using the Shapiro–Wilk test. Results of tests on normality of data are presented in Table S1. As most variables showed no significant deviation from normality, the dataset was assumed to follow a normal distribution for subsequent analyses.

To examine the relationship between skeletal asymmetry and soft tissue asymmetry, Pearson’s product–moment correlation coefficients (r) were calculated between each cephalometric parameter (independent variable) and the volumetric asymmetry of the midface or lower face (dependent variables). The coefficient of determination (R^2^) was obtained by squaring the Pearson correlation coefficients, which is mathematically equivalent to the value derived from simple linear regression.

All statistical analyses were performed using SPSS Statistics version 27.0 (IBM Japan, Tokyo, Japan). A *p*-value < 0.05 was considered statistically significant.

## 3. Results

### 3.1. Volumetric Soft Tissue Asymmetry

The mean volumetric asymmetry was 11.32 ± 8.06 cm^3^ for the whole face, 1.90 ± 3.08 cm^3^ for the midface, and 7.99 ± 5.99 cm^3^ for the lower face. Asymmetry was most pronounced in the lower face, followed by the whole face, and least in the midface. Figure 1 illustrates a representative case of volumetric deviation using mirror-image superimposition. Detailed descriptive statistics for all variables, including cephalometric and volumetric asymmetry measurements, are presented in Table 2.

### 3.2. Correlation Between Skeletal Asymmetry and Soft Tissue Asymmetry

**Figure 5:**
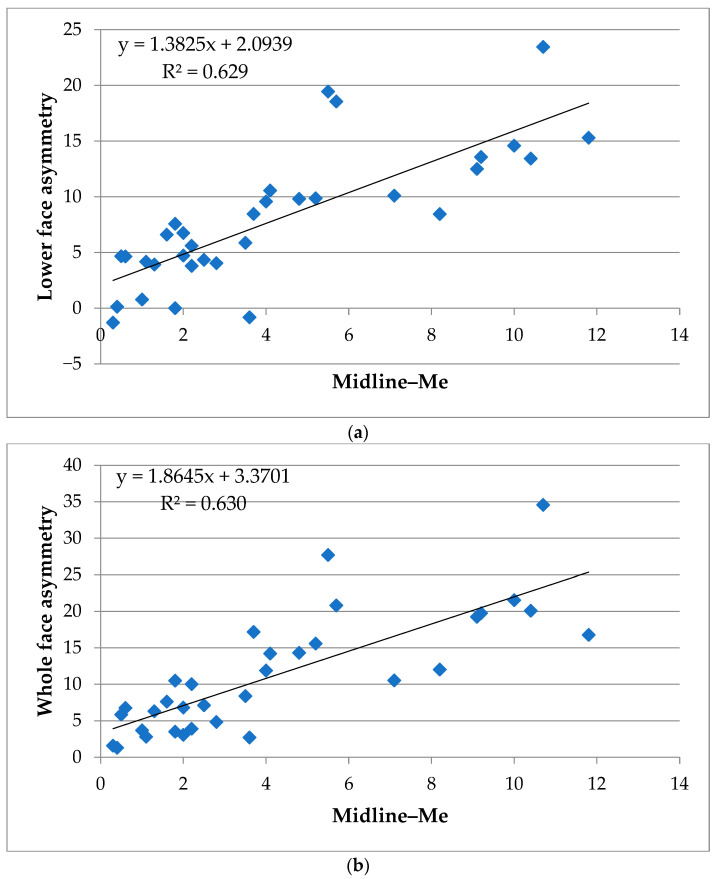
Scatter plots of skeletal and soft tissue asymmetry in the lower and whole face. (**a**) Correlation between lower facial soft tissue asymmetry volume and deviation of the menton from the midline (Midline–Me). (**b**) Correlation between whole facial soft tissue asymmetry volume and deviation of the menton from the midline (Midline–Me). Linear regression lines and coefficients of determination (R^2^) are displayed in each panel. Both plots highlight the strong association between menton deviation and volumetric soft tissue asymmetry in the lower and whole face regions.

**Figure 6:**
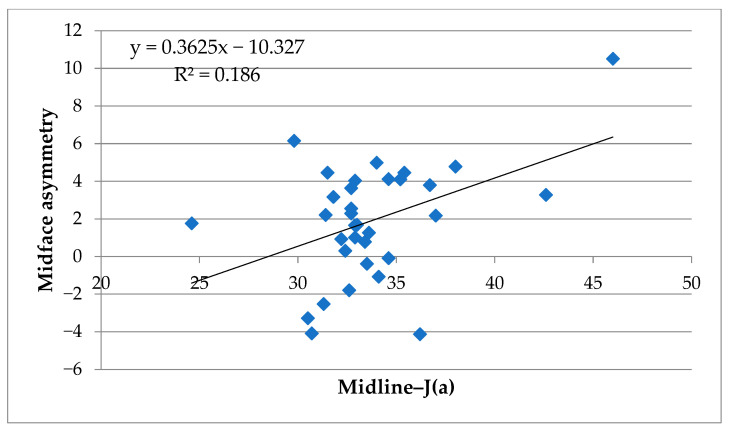
Scatter plot of skeletal and soft tissue asymmetry in the midface. Correlation between midfacial soft tissue asymmetry volume and deviation of J(a) from the midline (Midline–J(a)). The regression line and coefficient of determination (R^2^) are displayed.

The results of the correlation analyses between skeletal asymmetry parameters and volumetric soft tissue asymmetry are summarized in Table 3. To enhance readability and provide a more intuitive representation of the findings, key correlations were additionally visualized using scatter plots with regression lines and R^2^ values. Specifically, the strongest associations—(1) lower face asymmetry vs. Midline–Me, (2) whole face asymmetry vs. Midline–Me, and (3) midface asymmetry vs. Midline–J(a)—are presented in Figure 5 and Figure 6. These plots highlight the linear trends underlying the correlation coefficients and complement the numerical data presented in Table 3. A complete list of correlation coefficients (r, R^2^, and *p*-values) for all variables is provided in Supplementary Table S2.

#### 3.2.1. Lower Face

Among the 28 postero-anterior cephalometric variables, deviation of the menton from the midline (⑳ Midline–Me, R^2^ = 0.629 **) demonstrated the strongest correlation with lower facial soft tissue asymmetry. Other parameters showing substantial associations included ⑮ Midline–Mo diff (R^2^ = 0.295 **) and ㉘ ∠Mea (R^2^ = 0.292 **). Moderate associations were observed for ⑫ Midline–J diff (R^2^ = 0.226 *), ⑬ Midline–Mo(a) (R^2^ = 0.168 *), ⑭ Midline–Mo(b) (R^2^ = 0.159 *), ⑲ Go–Me diff (R^2^ = 0.187 *), ㉓ L6(a)–Midline (R^2^ = 0.237 *), and ㉔ L6(b)–Midline(R^2^ = 0.203 *).

#### 3.2.2. Whole Face

For the whole face, the strongest correlation was also observed for ⑳ Midline–Me (R^2^ = 0.630 **), followed by substantial associations for ⑮ Midline–Mo diff (R^2^ = 0.353 **), L6–Midline diff (R^2^ = 0.416 **), and ㉘ ∠Mea (R^2^ = 0.342 **). Moderate associations were found for ⑩ Midline–J(a) (R^2^ = 0.171 *), ⑫ Midline–J diff (R^2^ = 0.260 *), ⑬ Midline–Mo(a) (R^2^ = 0.180 *), ⑭ Midline–Mo(b) (R^2^ = 0.216 *), ⑲ Go–Me diff (R^2^ = 0.145 *), ㉓ L6(a)–Midline (R^2^ = 0.246 *), and ㉔ L6(b)–Midline (R^2^ = 0.262 *).

#### 3.2.3. Midface

In the midface, correlations with skeletal parameters were generally weaker. The highest values were for ⑩ Midline–J(a) (R^2^ = 0.186 *), classified as moderate associations. All other parameters showed weak (0.02 ≤ R^2^ < 0.13) or very weak (R^2^ < 0.02) relationships. As illustrated in Figure 6, the correlations in the midface region were weaker overall compared to those observed in the lower and whole face (Figure 5); however, the moderate association observed for Midline–J(a) indicates that midfacial skeletal asymmetry is not entirely unrelated to soft tissue asymmetry and warrants cautious interpretation.

### 3.3. Summary of Effect Sizes

Table 3 summarizes the coefficients of determination (R^2^) for each of the 28 cephalometric variables with respect to the volumetric asymmetry of the whole face, lower face, and midface. Following Cohen’s (1988) [41] guidelines for effect size in linear regression (R^2^ < 0.02: very weak; 0.02 ≤ R^2^ < 0.13: weak; 0.13 ≤ R^2^ < 0.26: moderate; R^2^ ≥ 0.26: substantial) [41], variables with moderate associations are indicated with an asterisk (*) and those with substantial associations are indicated with two asterisks (**). The overall pattern indicated that skeletal asymmetry—particularly menton deviation (⑳ Midline–Me)—had the strongest impact on lower and whole facial soft tissue asymmetry, whereas associations with midfacial asymmetry were relatively weaker.

In summary, scatter plot visualizations (Figure 5 and Figure 6) clearly demonstrate that skeletal asymmetry—particularly menton deviation (⑳ Midline–Me)—had the strongest impact on lower and whole facial soft tissue asymmetry, whereas associations with midfacial asymmetry were relatively weaker. Supplementary Table S2 provides a full correlation matrix including Pearson’s r, R^2^, and *p*-values for transparency.

## 4. Discussion

This study investigated the relationship between skeletal asymmetry measured on postero-anterior (PA) cephalometric radiographs and volumetric soft tissue asymmetry assessed via 3D stereophotogrammetry in patients with skeletal facial asymmetry. Our findings revealed a significant correlation between mandibular skeletal deviation—particularly menton deviation from the midline—and lower facial soft tissue asymmetry. In contrast, skeletal variables related to the midface showed weak or negligible correlations with midfacial soft tissue asymmetry. These results underscore the dominant influence of mandibular deviation on lower facial soft tissue imbalance. As a cross-sectional, single-center pilot investigation conducted in a treatment-seeking cohort, the present design was intended to identify candidate skeletal predictors within a clinically relevant population; nevertheless, the absence of a normative control group limits inferences about population-level thresholds and baseline variability.

Specifically, the strong correlation observed between the Midline–Me value and soft tissue asymmetry volume in the lower face (R^2^ = 0.629) supports previous reports that menton deviation is a reliable indicator of facial asymmetry severity [8,28,30]. Additional skeletal landmarks such as Midline–Mo diff and ∠Mea also demonstrated substantial associations (R^2^ = 0.295 and 0.292, respectively), suggesting that lower facial morphology is tightly linked to underlying skeletal structure [8,29].

In contrast, the weak correlations found in the midface suggest that midfacial soft tissue asymmetry is not strongly dictated by skeletal discrepancies detectable on PA cephalometry. This may be attributed to several factors. First, the midfacial region contains more abundant soft tissue padding, including the buccal fat pad and muscles of facial expression, which may obscure underlying skeletal asymmetry [1,2,25]. Second, midfacial structures such as the maxilla and nasal complex are less mobile and less variable in expression compared to the mandible, reducing the likelihood of external asymmetry despite internal skeletal deviations [26,27]. Nevertheless, a moderate association for Midline–J(a) was observed (R^2^ = 0.186), indicating that midfacial skeletal asymmetry is not entirely unrelated to soft tissue morphology. Accordingly, the interpretation of midfacial results should be made with caution rather than concluding a uniformly poor correlation. Moreover, functional factors such as the hypertrophy of masticatory muscles and lifestyle habits (e.g., habitual unilateral chewing) may independently contribute to soft tissue asymmetry, even in patients with relatively symmetrical skeletal morphology. These confounding influences were not assessed in the present study, and they should be recognized as potential contributors to lower facial imbalance beyond skeletal deviation. Future investigations incorporating functional assessments, such as electromyographic activity or masticatory habit questionnaires, will be valuable for clarifying the interplay between muscle function and soft tissue morphology. In addition, the choice of cephalometric midline definition may have influenced the results. In this study, the reference line connecting crista galli and U1 was used in accordance with previous PA cephalometric analyses of facial asymmetry [39]. While this method provides practical reproducibility, it carries the limitation that maxillary skeletal asymmetry can shift the incisor midpoint and thereby reduce the apparent discrepancy between midfacial skeletal and soft tissue landmarks. This potential underestimation should be acknowledged as a methodological limitation. Future studies should incorporate alternative reference planes (e.g., CG–ANS line) and three-dimensional skeletal analyses (e.g., CBCT) to improve anatomical accuracy and validity [38].

Another methodological aspect to consider is the definition of the midsagittal plane. In this study, we adopted the approach of Ueda et al. [35], defining the sagittal plane through Se and Sn, perpendicular to the coronal plane. This plane construction has been widely applied in stereophotogrammetric studies of facial asymmetry and provides anatomical consistency across patients. The reliability of this approach is further supported by validation studies of the VECTRA^®^ H1, which demonstrated excellent accuracy, repeatability, and reproducibility for midline-based facial measurements [33,34]. Therefore, the observed associations between skeletal and soft tissue asymmetry can be interpreted with confidence that they are not artifacts of plane definition or measurement error.

Our results are consistent with previous studies indicating that while mandibular asymmetry often manifests clearly in the soft tissue profile, midfacial asymmetry may be either masked or compensated by soft tissue dynamics [25,27,28]. For example, Uesugi et al. showed that a frontal occlusal plane inclined contralaterally to the mandibular deviation is associated with evident lower-facial imbalance, underscoring the dominant effect of mandibular deviation on soft-tissue asymmetry [28]. Moreover, other reports have suggested that maxillary cant and transverse discrepancies in the midface may require more precise imaging modalities, such as CBCT, for accurate correlation with soft tissue changes [16,30,31]. In addition, many of the skeletal indices analyzed in the present study were not specific to the midfacial region (e.g., measures not directly capturing maxillary cant, transverse discrepancies, or nasal skeletal morphology). This lack of region-specific parameters likely contributed to the relatively weak associations observed in the midface. Future studies should incorporate midface-specific skeletal indices and three-dimensional skeletal assessments (e.g., CBCT-based measures) to better elucidate the structural determinants of midfacial soft tissue asymmetry.

Another important consideration is the regional independence of facial asymmetry. While it is common to conceptualize facial asymmetry as a unified deformity, our findings highlight that lower and midfacial asymmetries may have distinct etiologies and clinical implications. This underscores the importance of region-specific evaluation and treatment planning. For instance, surgical correction of mandibular deviation may significantly improve lower facial asymmetry but have limited impact on midfacial balance unless maxillary asymmetries are concurrently addressed [8,28,29].

There are several limitations to this study. First, the sample consisted exclusively of Japanese patients undergoing orthognathic surgery for skeletal facial asymmetry, which may limit generalizability to other populations and skeletal types. Second, the study did not include a nonsurgical control group with “normal” skeletal parameters within the same ethnic background. The lack of sex- and age-matched controls prevents direct benchmarking of volumetric soft tissue asymmetry and may bias effect-size estimates (either inflating or attenuating correlations) relative to normative variability. Third, the evaluation of asymmetry was limited to static conditions without consideration of facial animation or functional movement, which may reveal additional dimensions of asymmetry not captured in resting scans [25,32]. Fourth, while PA cephalometry provides a convenient and widely used method for skeletal assessment, its 2D nature and susceptibility to projection errors may restrict its accuracy in detecting complex midfacial asymmetries [9,31,38]. Fifth, a substantial proportion of the present cohort exhibited relatively symmetrical soft tissue morphology in the midfacial region. This is likely attributable to the case composition at our institution, where mandibular deviation is the predominant indication for orthognathic surgery. Such a sample characteristic may have led to an underrepresentation of patients with pronounced midfacial soft tissue asymmetry, thereby contributing to the weak or insignificant correlations observed between skeletal and midfacial soft tissue variables. This potential selection bias should be considered when interpreting the findings. Sixth, the set of 3D soft tissue indices analyzed in this study was relatively limited. We quantified volumetric asymmetry using mirrored surface superimposition, but this approach does not differentiate asymmetry by spatial direction. In clinical practice, discrepancies along the sagittal dimension (anteroposterior) versus the coronal plane (transverse) may have distinct implications for diagnosis and surgical correction. Therefore, the lack of directional analysis represents a methodological limitation that may have masked clinically relevant patterns. Future research should incorporate directional or vector-based 3D asymmetry indices to capture the orientation of discrepancies and improve their clinical interpretability. To address the control-group limitation, future studies will recruit a matched normative cohort (women and men) from the same ethnic population and apply stratified or propensity score–matched analyses to compare (i) absolute volumetric asymmetry, (ii) region-specific distributions, and (iii) the predictive value of skeletal parameters across clinical vs. normative samples. Where prospective recruitment is not feasible, we will leverage available normative databases to derive age- and sex-adjusted reference intervals and sensitivity analyses.

Future research should incorporate a greater number of patients with clinically evident midfacial asymmetry to clarify whether specific skeletal discrepancies—such as maxillary cant or transverse deviations—underlie soft tissue imbalance in this region. Furthermore, to address the limitation of parameter selection, future work will prioritize the inclusion of midface-specific skeletal metrics (e.g., maxillary cant, transverse maxillary width, nasal skeletal parameters) and leverage CBCT-based 3D analyses aligned with the stereophotogrammetric surface to enable more anatomically targeted correlations.

Although the present study found limited associations between midfacial skeletal measurements and soft tissue asymmetry, this may be partly attributable to the relatively small proportion of patients with pronounced midfacial soft tissue asymmetry in the current sample. Future research should include a greater number of cases exhibiting midfacial soft tissue asymmetry in order to examine whether specific skeletal features—such as maxillary cant or transverse discrepancies—underlie midfacial soft tissue imbalance. Furthermore, investigating the correlations between midfacial and lower facial soft tissue asymmetry may offer insights into the structural interplay between facial regions in patients with skeletal Class III or asymmetric deformities.

In the future, artificial intelligence (AI) and machine learning-based analysis of 3D facial images are expected to play an important role in the diagnosis and treatment prediction of facial asymmetry. In particular, AI-driven automated region segmentation and asymmetry quantification can enhance the objectivity and reproducibility of assessments and can also be useful for building predictive models of postoperative changes [7,36]. Furthermore, integrating surface electromyography (sEMG) with 3D dynamic analysis would enable a comprehensive diagnosis that incorporates not only static evaluation but also dynamic functional assessment [43]. These technologies are considered likely to contribute to the advancement of individualized treatment planning and the prediction of surgical outcomes.

These findings highlight the importance of region-specific assessment in the diagnosis and treatment planning of patients with facial asymmetry. Cephalometric indicators such as Midline–Me and mandibular body asymmetry may serve as reliable predictors of lower facial imbalance, whereas midfacial asymmetry requires further investigation using more sensitive or three-dimensional skeletal evaluation methods. Further studies involving patients with pronounced midfacial asymmetry are warranted to elucidate the skeletal determinants of soft tissue imbalance in the midface and its relationship to lower facial asymmetry.

## 5. Conclusions

This study demonstrated that volumetric soft tissue asymmetry in the lower face is strongly correlated with skeletal deviations of the mandible, particularly menton deviation from the facial midline. In contrast, soft tissue asymmetry in the midface showed only weak associations with cephalometric measurements, suggesting that midfacial skeletal asymmetry may be less visually expressed or more effectively compensated by overlying soft tissues.

## Figures and Tables

**Table 1:** Anatomical landmarks, reference lines, and cephalometric measurements used in this study.

No.	Landmark/Measurement	Definition
Landmarks		
CG	Crista galli	Most superior point of the crista galli in the midline
ANS	Anterior nasal spine	Most anterior point of the anterior nasal spine
Lo	Orbitale lateral point	Intersection of the lateral orbital rim and oblique line
J	Jugale	Intersection of the maxillary tuberosity and zygomatic arch
Mo	Maxillary first molar	Center of the maxillary first molar
U1	Upper central incisor	Midpoint of the maxillary central incisor
Go	Gonion	Most lateral point on the mandibular angle
Ag	Antegonial notch	Most concave point of the antegonial notch
Me	Menton	Most inferior point on the mandibular symphysis
Reference lines		
Midline	CG–ANS	Line connecting CG and ANS
Lo line	Lo(a)–Lo(b)	Line connecting bilateral Lo points
FP	Lo(a)–Ag(a)	Line from Lo on deviated side to Ag on same side
FP′	Lo(b)–Ag(b)	Line from Lo on contralateral side to Ag on same side
Linear measurements		
①	Lo line	Distance between Lo(a) and Lo(b)
②	FP(a)	Distance Lo(a)–Ag(a)
③	FP(b)	Distance Lo(b)–Ag(b)
④	Lo line–J(a)	Perpendicular distance from Lo line to J(a)
⑤	Lo line–J(b)	Perpendicular distance from Lo line to J(b)
⑥	Lo line–J diff	Difference between ④ and ⑤
⑦	Lo line–Mo(a)	Perpendicular distance from Lo line to Mo(a)
⑧	Lo line–Mo(b)	Perpendicular distance from Lo line to Mo(b)
⑨	Lo line–Mo diff	Difference between ⑦ and ⑧
⑩	Midline–J(a)	Distance from midline to J(a)
⑪	Midline–J(b)	Distance from midline to J(b)
⑫	Midline–J diff	Difference between ⑩ and ⑪
⑬	Midline–Mo(a)	Distance from midline to Mo(a)
⑭	Midline–Mo(b)	Distance from midline to Mo(b)
⑮	Midline–Mo diff	Difference between ⑬ and ⑭
⑯	Midline–U1	Distance from midline to U1
⑰	Go(a)–Me	Distance between Go(a) and Me
⑱	Go(b)–Me	Distance between Go(b) and Me
⑲	Go–Me diff	Difference between ⑰ and ⑱
⑳	Midline–Me	Distance from midline to Me
㉑	L6(a)–FP	Distance from upper first molar on deviated side to FP
㉒	L6(b)–FP′	Distance from upper first molar on contralateral side to FP′
㉓	L6(a)–Midline	Distance from upper first molar on deviated side to midline
㉔	L6(b)–Midline	Distance from upper first molar on contralateral side to midline
Angular measurements		
㉕	∠Fmp	Angle between Go(a)–Go(b) and perpendicular to midline
㉖	∠J	Angle between J(a)–J(b) and perpendicular to midline
㉗	∠Ocl	Angle between Mo(a)–Mo(b) and perpendicular to midline
㉘	∠Mea	Angle between ANS–Me and midline

**Table 2:** Descriptive statistics for cephalometric measurements.

No	Variable	Mean	95% CI Lower	95% CI Upper	SD	Min	Max
①	Lo line	93.35	92.05	94.65	3.67	87.40	101.90
②	FP(a)	97.62	95.35	99.88	6.39	85.30	109.30
③	FP(b)	98.85	96.71	101.00	6.04	87.40	112.40
④	Lo line–J(a)	61.15	59.28	63.01	5.25	50.40	70.40
⑤	Lo line–J(b)	61.92	59.93	63.92	5.63	49.60	72.30
⑥	Lo line–J diff	−0.78	−1.61	0.06	2.36	−6.50	5.80
⑦	Lo line–Mo(a)	81.64	79.72	83.57	5.43	70.40	93.80
⑧	Lo line–Mo(b)	83.39	81.44	85.34	5.50	70.90	95.40
⑨	Lo line–Mo diff	−1.75	−2.39	−1.10	1.82	−5.00	1.50
⑩	Midline–J(a)	33.74	32.44	35.04	3.66	24.60	46.00
⑪	Midline–J(b)	33.30	32.13	34.46	3.28	26.70	42.70
⑫	Midline–J diff	0.45	−0.43	1.32	2.47	−4.60	6.00
⑬	Midline–Mo(a)	24.66	23.65	25.67	2.84	18.60	30.00
⑭	Midline–Mo(b)	21.98	21.06	22.91	2.61	17.00	28.40
⑮	Midline–Mo diff	2.68	1.23	4.12	4.07	−5.80	12.00
⑯	Midline–U1	0.93	0.56	1.30	1.03	−1.00	3.20
⑰	Go(a)–Me	61.18	58.86	63.49	6.53	49.20	71.70
⑱	Go(b)–Me	65.37	63.37	67.36	5.63	55.40	74.90
⑲	Go–Me diff	−4.19	−5.65	−2.73	4.12	−14.70	2.40
⑳	Midline–Me	4.26	3.05	5.48	3.43	0.30	11.80
㉑	L6(a)–FP	24.67	23.33	26.01	3.77	17.40	31.40
㉒	L6(b)–FP’	24.46	23.58	25.35	2.50	19.40	29.10
㉓	L6(a)–Midline	23.82	22.70	24.95	3.17	16.80	30.60
㉔	L6(b)–Midline	21.01	20.03	21.98	2.75	13.30	26.20
㉕	∠Fmp	1.88	1.13	2.63	2.12	0.00	9.00
㉖	∠J	1.64	1.07	2.20	1.59	0.00	7.00
㉗	∠Ocl	1.94	1.10	2.78	2.36	0.00	9.00
㉘	∠Mea	3.68	2.92	4.44	2.14	0.00	8.00

**Table 3:** Coefficients of determination (R^2^) between cephalometric variables and volumetric asymmetry of the whole face, lower face, and midface.

No	Variable	R^2^ Whole Face	R^2^ Lower Face	R^2^ Midface
①	Lo line	0.004	<0.001	0.005
②	FP(a)	<0.001	0.003	0.000
③	FP(b)	0.005	<0.001	0.006
④	Lo line–J(a)	0.011	0.007	0.001
⑤	Lo line–J(b)	0.004	0.003	0.000
⑥	Lo line–J diff	0.007	0.003	0.002
⑦	Lo line–Mo(a)	0.009	0.004	0.008
⑧	Lo line–Mo(b)	<0.001	0.002	0.004
⑨	Lo line–Mo diff	0.095	0.106	0.008
⑩	Midline–J(a)	0.171 *	0.125	0.186 *
⑪	Midline–J(b)	0.006	0.001	0.052
⑫	Midline–J diff	0.260 **	0.226 *	0.112
⑬	Midline–Mo(a)	0.180 *	0.168 *	0.015
⑭	Midline–Mo(b)	0.216 *	0.159 *	0.041
⑮	Midline–Mo diff	0.353 **	0.295 **	0.046
⑯	Midline–U1	0.077	0.115	0.001
⑰	Go(a)–Me	0.021	0.041	0.012
⑱	Go(b)–Me	0.012	0.007	0.028
⑲	Go–Me diff	0.145 *	0.187 *	0.003
⑳	Midline–Me	0.630 **	0.629 **	0.129
㉑	L6(a)–FP	0.003	0.001	0.011
㉒	L6(b)–FP’	0.010	0.013	0.000
㉓	L6(a)–Midline	0.246 *	0.237 *	0.029
㉔	L6(b)–Midline	0.262 **	0.203 *	0.061
㉕	∠Fmp	0.035	0.034	0.002
㉖	∠J	0.015	0.019	0.014
㉗	∠Ocl	0.003	<0.001	0.000
㉘	∠Mea	0.342 **	0.292 **	0.036

(* = moderate [0.13 ≤ R^2^ < 0.26], ** = substantial [R^2^ ≥ 0.26], Cohen 1988 [41]).

## Data Availability

All of clinical data are available in the Supplementary Materials.

## Supplementary Materials

The following supporting information can be downloaded at: https://www.mdpi.com/article/10.3390/jcm14196721/s1, Table S1: Results of tests on normality of data, Table S2: Correlation coefficients between cephalometric variables and volumetric asymmetry subdivided by (a) whole face, (b) lower face, and (c) midface, File S1: All the data analyzed in this study.
