# Supplementary material for: Correlation Between Volumetric Soft Tissue Asymmetry and Postero-Anterior Cephalometric Measurements in Patients with Skeletal Facial Asymmetry: A Cross-Sectional Pilot Study
Source: J Clin Med. 2025 Sep 23;14(19):6721. doi: 10.3390/jcm14196721 (PMC12525259; doi:10.3390/jcm14196721)
Supplement: Supplementary file 1 [file jcm-14-06721-s001.zip › Table S1,S2.pptx]

## Slide 1
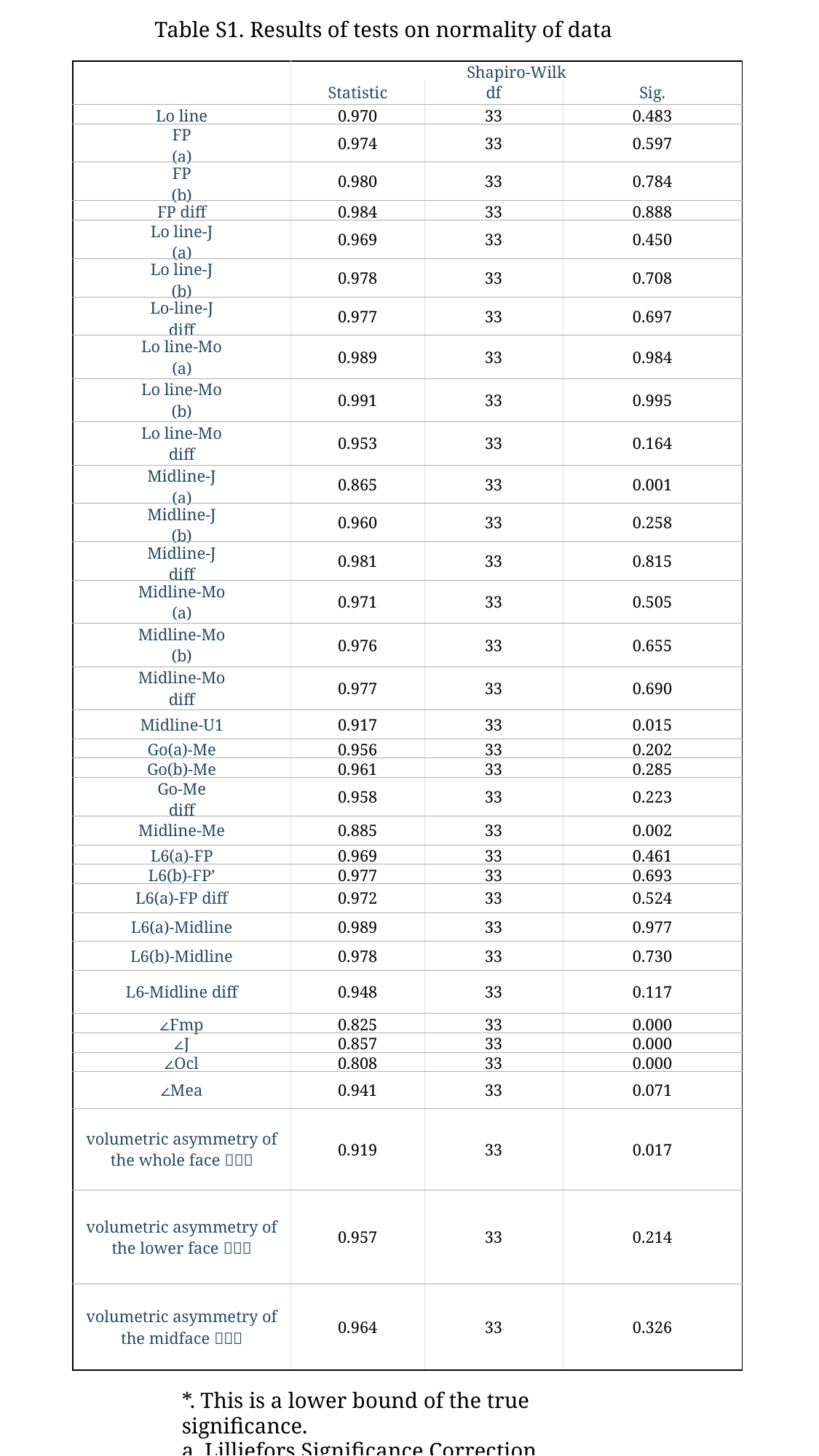

Table S1. Results of tests on normality of data
| | Shapiro-Wilk | | |
| --- | --- | --- | --- |
| | Statistic | df | Sig. |
| Lo line | 0.970 | 33 | 0.483 |
| FP (a) | 0.974 | 33 | 0.597 |
| FP (b) | 0.980 | 33 | 0.784 |
| FP diff | 0.984 | 33 | 0.888 |
| Lo line-J (a) | 0.969 | 33 | 0.450 |
| Lo line-J (b) | 0.978 | 33 | 0.708 |
| Lo-line-J diff | 0.977 | 33 | 0.697 |
| Lo line-Mo (a) | 0.989 | 33 | 0.984 |
| Lo line-Mo (b) | 0.991 | 33 | 0.995 |
| Lo line-Mo diff | 0.953 | 33 | 0.164 |
| Midline-J (a) | 0.865 | 33 | 0.001 |
| Midline-J (b) | 0.960 | 33 | 0.258 |
| Midline-J diff | 0.981 | 33 | 0.815 |
| Midline-Mo (a) | 0.971 | 33 | 0.505 |
| Midline-Mo (b) | 0.976 | 33 | 0.655 |
| Midline-Mo diff | 0.977 | 33 | 0.690 |
| Midline-U1 | 0.917 | 33 | 0.015 |
| Go(a)-Me | 0.956 | 33 | 0.202 |
| Go(b)-Me | 0.961 | 33 | 0.285 |
| Go-Me diff | 0.958 | 33 | 0.223 |
| Midline-Me | 0.885 | 33 | 0.002 |
| L6(a)-FP | 0.969 | 33 | 0.461 |
| L6(b)-FP’ | 0.977 | 33 | 0.693 |
| L6(a)-FP diff | 0.972 | 33 | 0.524 |
| L6(a)-Midline | 0.989 | 33 | 0.977 |
| L6(b)-Midline | 0.978 | 33 | 0.730 |
| L6-Midline diff | 0.948 | 33 | 0.117 |
| ∠Fmp | 0.825 | 33 | 0.000 |
| ∠J | 0.857 | 33 | 0.000 |
| ∠Ocl | 0.808 | 33 | 0.000 |
| ∠Mea | 0.941 | 33 | 0.071 |
| volumetric asymmetry of the whole face（㎤） | 0.919 | 33 | 0.017 |
| volumetric asymmetry of the lower face（㎤） | 0.957 | 33 | 0.214 |
| volumetric asymmetry of the midface（㎤） | 0.964 | 33 | 0.326 |
*. This is a lower bound of the true significance.
a. Lilliefors Significance Correction

## Slide 2
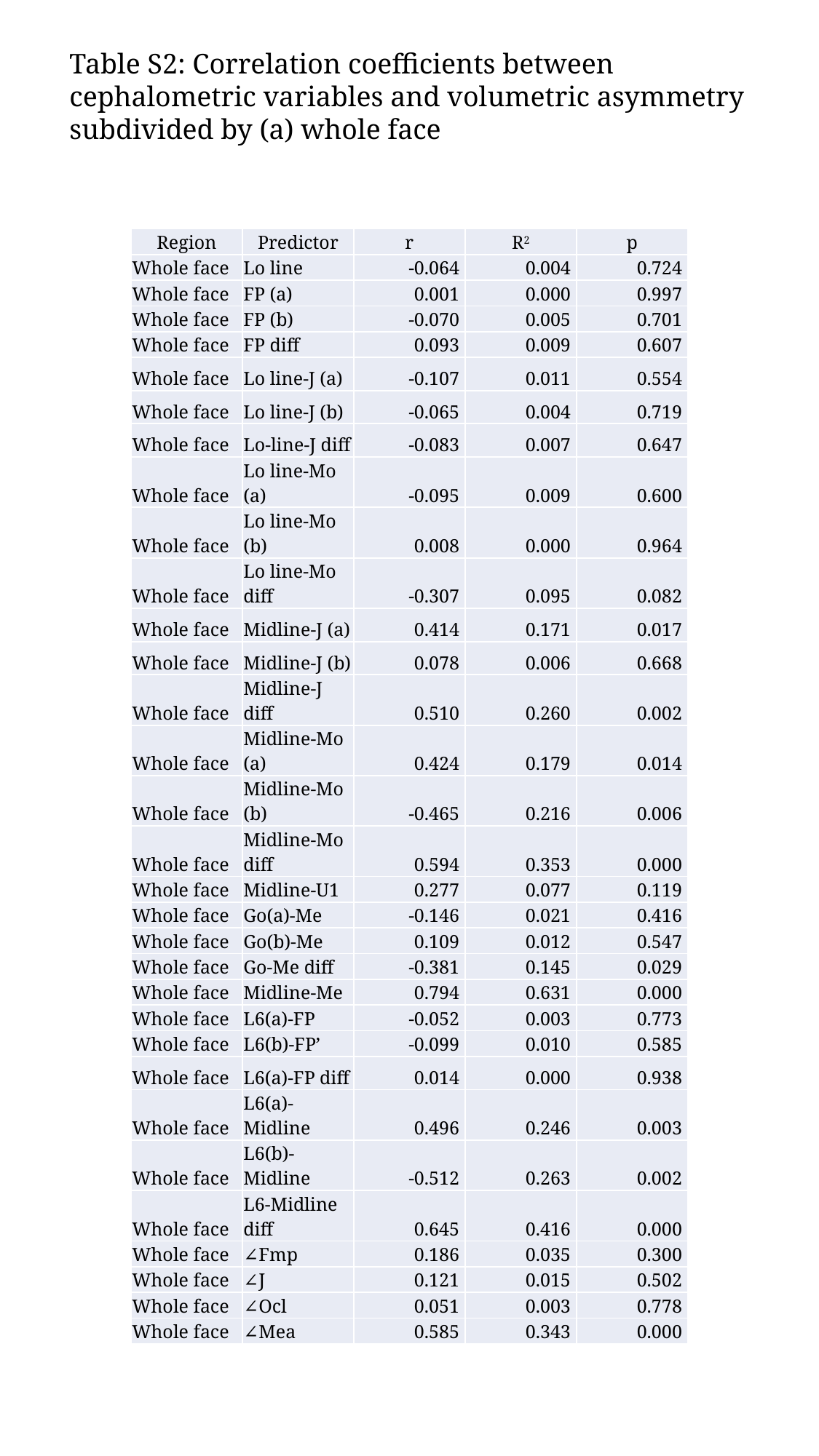

Table S2: Correlation coefficients between cephalometric variables and volumetric asymmetry subdivided by (a) whole face
| Region | Predictor | r | R2 | p |
| --- | --- | --- | --- | --- |
| Whole face | Lo line | -0.064 | 0.004 | 0.724 |
| Whole face | FP (a) | 0.001 | 0.000 | 0.997 |
| Whole face | FP (b) | -0.070 | 0.005 | 0.701 |
| Whole face | FP diff | 0.093 | 0.009 | 0.607 |
| Whole face | Lo line-J (a) | -0.107 | 0.011 | 0.554 |
| Whole face | Lo line-J (b) | -0.065 | 0.004 | 0.719 |
| Whole face | Lo-line-J diff | -0.083 | 0.007 | 0.647 |
| Whole face | Lo line-Mo (a) | -0.095 | 0.009 | 0.600 |
| Whole face | Lo line-Mo (b) | 0.008 | 0.000 | 0.964 |
| Whole face | Lo line-Mo diff | -0.307 | 0.095 | 0.082 |
| Whole face | Midline-J (a) | 0.414 | 0.171 | 0.017 |
| Whole face | Midline-J (b) | 0.078 | 0.006 | 0.668 |
| Whole face | Midline-J diff | 0.510 | 0.260 | 0.002 |
| Whole face | Midline-Mo (a) | 0.424 | 0.179 | 0.014 |
| Whole face | Midline-Mo (b) | -0.465 | 0.216 | 0.006 |
| Whole face | Midline-Mo diff | 0.594 | 0.353 | 0.000 |
| Whole face | Midline-U1 | 0.277 | 0.077 | 0.119 |
| Whole face | Go(a)-Me | -0.146 | 0.021 | 0.416 |
| Whole face | Go(b)-Me | 0.109 | 0.012 | 0.547 |
| Whole face | Go-Me diff | -0.381 | 0.145 | 0.029 |
| Whole face | Midline-Me | 0.794 | 0.631 | 0.000 |
| Whole face | L6(a)-FP | -0.052 | 0.003 | 0.773 |
| Whole face | L6(b)-FP’ | -0.099 | 0.010 | 0.585 |
| Whole face | L6(a)-FP diff | 0.014 | 0.000 | 0.938 |
| Whole face | L6(a)-Midline | 0.496 | 0.246 | 0.003 |
| Whole face | L6(b)-Midline | -0.512 | 0.263 | 0.002 |
| Whole face | L6-Midline diff | 0.645 | 0.416 | 0.000 |
| Whole face | ∠Fmp | 0.186 | 0.035 | 0.300 |
| Whole face | ∠J | 0.121 | 0.015 | 0.502 |
| Whole face | ∠Ocl | 0.051 | 0.003 | 0.778 |
| Whole face | ∠Mea | 0.585 | 0.343 | 0.000 |

## Slide 3
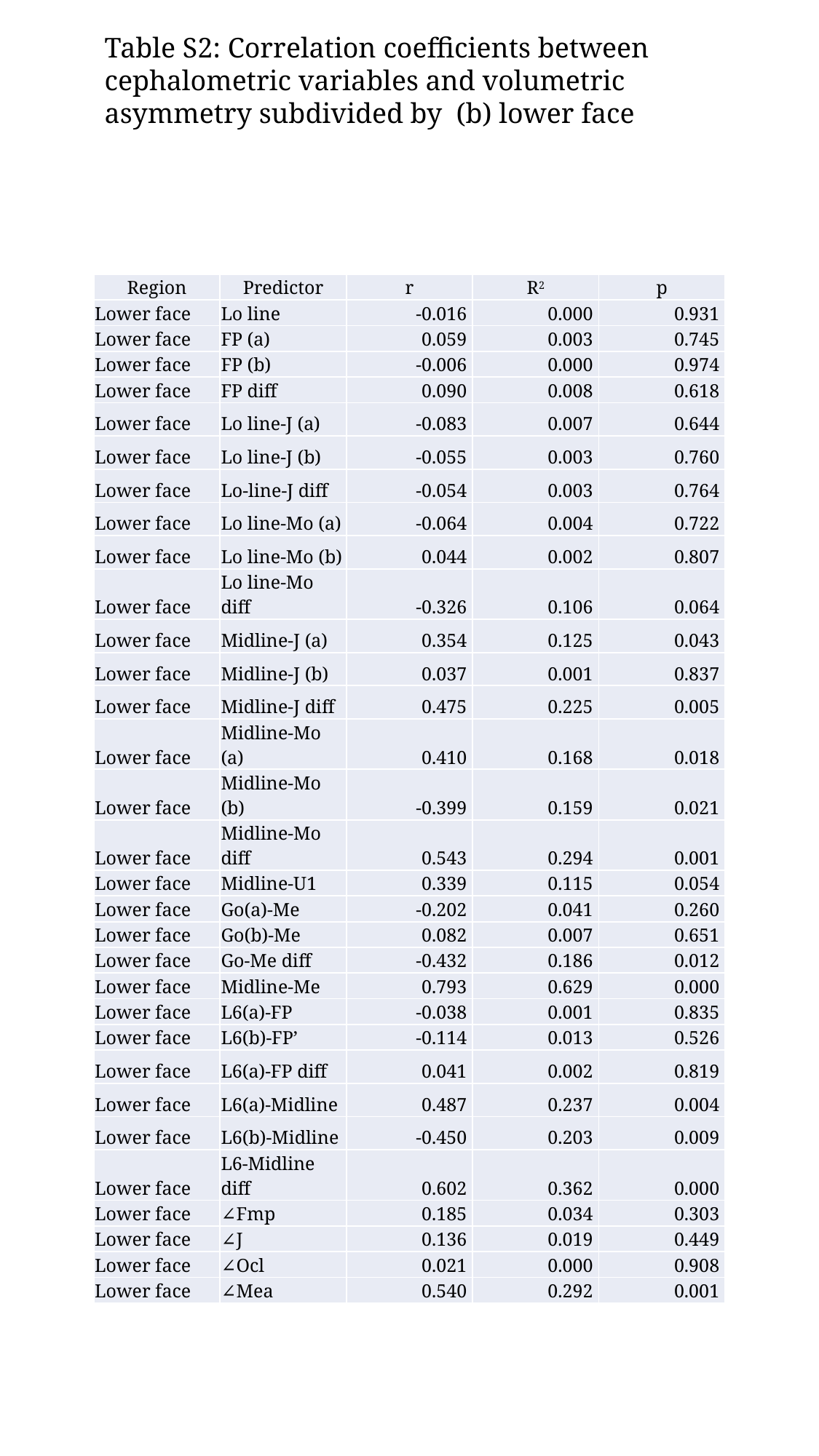

Table S2: Correlation coefficients between cephalometric variables and volumetric asymmetry subdivided by (b) lower face
| Region | Predictor | r | R2 | p |
| --- | --- | --- | --- | --- |
| Lower face | Lo line | -0.016 | 0.000 | 0.931 |
| Lower face | FP (a) | 0.059 | 0.003 | 0.745 |
| Lower face | FP (b) | -0.006 | 0.000 | 0.974 |
| Lower face | FP diff | 0.090 | 0.008 | 0.618 |
| Lower face | Lo line-J (a) | -0.083 | 0.007 | 0.644 |
| Lower face | Lo line-J (b) | -0.055 | 0.003 | 0.760 |
| Lower face | Lo-line-J diff | -0.054 | 0.003 | 0.764 |
| Lower face | Lo line-Mo (a) | -0.064 | 0.004 | 0.722 |
| Lower face | Lo line-Mo (b) | 0.044 | 0.002 | 0.807 |
| Lower face | Lo line-Mo diff | -0.326 | 0.106 | 0.064 |
| Lower face | Midline-J (a) | 0.354 | 0.125 | 0.043 |
| Lower face | Midline-J (b) | 0.037 | 0.001 | 0.837 |
| Lower face | Midline-J diff | 0.475 | 0.225 | 0.005 |
| Lower face | Midline-Mo (a) | 0.410 | 0.168 | 0.018 |
| Lower face | Midline-Mo (b) | -0.399 | 0.159 | 0.021 |
| Lower face | Midline-Mo diff | 0.543 | 0.294 | 0.001 |
| Lower face | Midline-U1 | 0.339 | 0.115 | 0.054 |
| Lower face | Go(a)-Me | -0.202 | 0.041 | 0.260 |
| Lower face | Go(b)-Me | 0.082 | 0.007 | 0.651 |
| Lower face | Go-Me diff | -0.432 | 0.186 | 0.012 |
| Lower face | Midline-Me | 0.793 | 0.629 | 0.000 |
| Lower face | L6(a)-FP | -0.038 | 0.001 | 0.835 |
| Lower face | L6(b)-FP’ | -0.114 | 0.013 | 0.526 |
| Lower face | L6(a)-FP diff | 0.041 | 0.002 | 0.819 |
| Lower face | L6(a)-Midline | 0.487 | 0.237 | 0.004 |
| Lower face | L6(b)-Midline | -0.450 | 0.203 | 0.009 |
| Lower face | L6-Midline diff | 0.602 | 0.362 | 0.000 |
| Lower face | ∠Fmp | 0.185 | 0.034 | 0.303 |
| Lower face | ∠J | 0.136 | 0.019 | 0.449 |
| Lower face | ∠Ocl | 0.021 | 0.000 | 0.908 |
| Lower face | ∠Mea | 0.540 | 0.292 | 0.001 |

## Slide 4
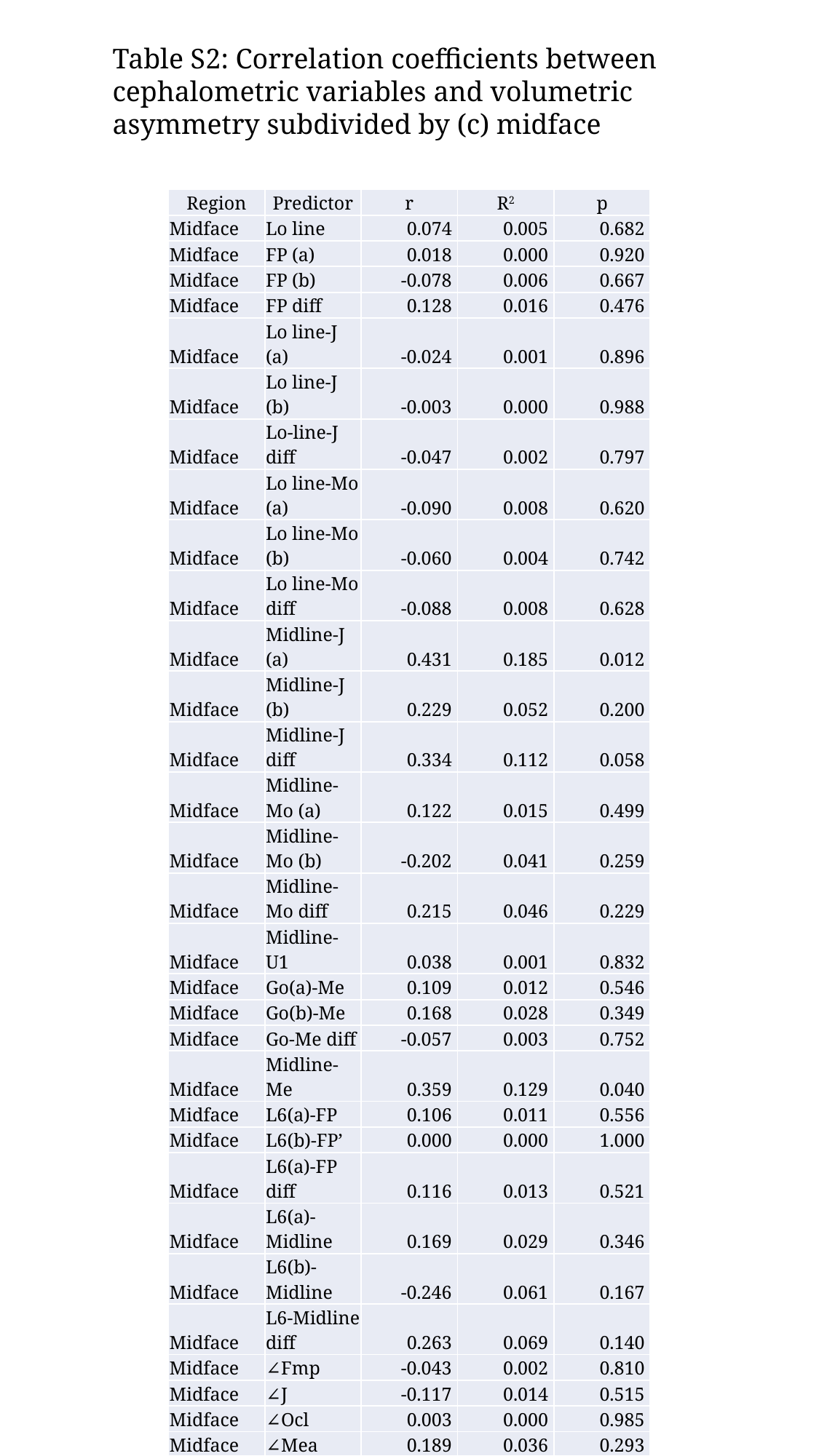

Table S2: Correlation coefficients between cephalometric variables and volumetric asymmetry subdivided by (c) midface
| Region | Predictor | r | R2 | p |
| --- | --- | --- | --- | --- |
| Midface | Lo line | 0.074 | 0.005 | 0.682 |
| Midface | FP (a) | 0.018 | 0.000 | 0.920 |
| Midface | FP (b) | -0.078 | 0.006 | 0.667 |
| Midface | FP diff | 0.128 | 0.016 | 0.476 |
| Midface | Lo line-J (a) | -0.024 | 0.001 | 0.896 |
| Midface | Lo line-J (b) | -0.003 | 0.000 | 0.988 |
| Midface | Lo-line-J diff | -0.047 | 0.002 | 0.797 |
| Midface | Lo line-Mo (a) | -0.090 | 0.008 | 0.620 |
| Midface | Lo line-Mo (b) | -0.060 | 0.004 | 0.742 |
| Midface | Lo line-Mo diff | -0.088 | 0.008 | 0.628 |
| Midface | Midline-J (a) | 0.431 | 0.185 | 0.012 |
| Midface | Midline-J (b) | 0.229 | 0.052 | 0.200 |
| Midface | Midline-J diff | 0.334 | 0.112 | 0.058 |
| Midface | Midline-Mo (a) | 0.122 | 0.015 | 0.499 |
| Midface | Midline-Mo (b) | -0.202 | 0.041 | 0.259 |
| Midface | Midline-Mo diff | 0.215 | 0.046 | 0.229 |
| Midface | Midline-U1 | 0.038 | 0.001 | 0.832 |
| Midface | Go(a)-Me | 0.109 | 0.012 | 0.546 |
| Midface | Go(b)-Me | 0.168 | 0.028 | 0.349 |
| Midface | Go-Me diff | -0.057 | 0.003 | 0.752 |
| Midface | Midline-Me | 0.359 | 0.129 | 0.040 |
| Midface | L6(a)-FP | 0.106 | 0.011 | 0.556 |
| Midface | L6(b)-FP’ | 0.000 | 0.000 | 1.000 |
| Midface | L6(a)-FP diff | 0.116 | 0.013 | 0.521 |
| Midface | L6(a)-Midline | 0.169 | 0.029 | 0.346 |
| Midface | L6(b)-Midline | -0.246 | 0.061 | 0.167 |
| Midface | L6-Midline diff | 0.263 | 0.069 | 0.140 |
| Midface | ∠Fmp | -0.043 | 0.002 | 0.810 |
| Midface | ∠J | -0.117 | 0.014 | 0.515 |
| Midface | ∠Ocl | 0.003 | 0.000 | 0.985 |
| Midface | ∠Mea | 0.189 | 0.036 | 0.293 |
